# Paired maternal and fetal metabolomics reveal a differential fingerprint in preeclampsia versus fetal growth restriction

**DOI:** 10.1038/s41598-021-93936-9

**Published:** 2021-07-13

**Authors:** Lina Youssef, Rui V. Simões, Jezid Miranda, María Luisa García-Martín, Cristina Paules, Francesca Crovetto, Nuria Amigó, Nicolau Cañellas, Eduard Gratacos, Fatima Crispi

**Affiliations:** 1 BCNatal|Fetal Medicine Research Center (Hospital Clínic and Hospital Sant Joan de Déu), Institut D’Investigacions Biomèdiques August Pi I Sunyer (IDIBAPS), University of Barcelona, Barcelona, Spain; 2 BIONAND, Andalusian Centre for Nanomedicine and Biotechnology (Junta de Andalucía- Universidad de Málaga), Málaga, Spain; 3 Biosfer Teslab, Reus, Spain; 4 Department of Basic Medical Sciences, University Rovira I Virgili, CIBERDEM, Reus, Spain; 5 Universidad Rovira I Virgili, DEEEiA, IISPV, Tarragona, Spain; 6 CIBERDEM, Spanish Biomedical Research Centre in Diabetes and Associated Metabolic Disorders, Madrid, Spain; 7 Centre for Biomedical Research on Rare Diseases (CIBER-ER), Madrid, Spain; 8 Champalimaud Research, Champalimaud Centre for the Unknown, Av Brasília, 1400-038 Lisbon, Portugal

**Keywords:** Metabolomics, Pre-eclampsia

## Abstract

Preeclampsia (PE) and fetal growth restriction (FGR) are both placenta-mediated disorders with unclear pathogenesis. Metabolomics of maternal and fetal pairs might help in understanding these disorders. We recruited prospectively pregnancies with normotensive FGR, PE without FGR, PE + FGR and uncomplicated pregnancies as controls. Nuclear magnetic resonance metabolomics were applied on plasma samples collected at delivery. Advanced lipoprotein, glycoprotein and choline profiling was performed using the Liposcale test. The software package Dolphin was used to quantify 24 low-molecular-weight metabolites. Statistical analysis comprised the comparison between each group of complicated pregnancies versus controls, considering 5% false discovery rate correction. Lipid profiles were altered in accordance with the clinical presentation of these disorders. Specifically, PE mothers and FGR fetuses (with or without FGR or PE, respectively) exhibited a pro-atherogenic and pro-inflammatory profile, with higher concentrations of triglycerides, remnant cholesterol (VLDL, IDL) and Glc/GalNAc-linked and lipid-associated glycoproteins compared to controls. Low-molecular-weight metabolites were extensively disturbed in preeclamptic mothers, with or without FGR. Growth restricted fetuses in the presence of PE showed changes in low-molecular-weight metabolites similar to their mothers (increased creatine and creatinine), while normotensive FGR fetuses presented scarce differences, consistent with undernutrition (lower isoleucine). Further research is warranted to clarify maternal and fetal adaptations to PE and FGR.

## Introduction

Preeclampsia (PE) and fetal growth restriction (FGR) are two common pregnancy-specific conditions with a recognized ominous effect on the mother and the developing fetus^1,2^. PE and FGR affect 2–8% and 5–10% of all pregnancies, respectively, and represent a major cause of maternal and perinatal morbidity and mortality, adding up long-term consequences on the cardiovascular, neurological and metabolic functions^1–4^. Both syndromes are considered placenta-mediated disorders since their pathophysiological basis is similar, with placental dysfunction from early pregnancy stages being the main culprit^5,6^. Thus, it is not surprising that these two disorders may co-exist, especially in early-onset cases that constitute the most severe clinical presentation in both cases. Efforts have been made to unveil the mechanistic pathways involved in maternal and fetal response to placenta-mediated disorders. However, it is still unclear how PE and FGR develop and affect mothers and fetuses. Understanding the specific alterations that occur in PE and FGR could be paramount for improving the clinical management of these disorders.

Metabolomics, the youngest of ‘Omics’ technologies, provides a fingerprinting of the metabolite distribution in a biological sample, as a reflex of the underlying biochemical reactions^7^. Metabolome analysis has strong potential for detailed phenotyping of PE and FGR, and has been applied on different pregnancy samples collected from the mother, and to a lesser extent, from the fetus^8,9^. To date, a handful of studies have focused on women with active disease, reporting metabolic patterns attributed to lipid metabolism^10^ and series of low-molecular-weight metabolites as potential biomarkers for PE^11–13^ or portraying the metabolic status of growth restricted fetuses^14–17^*.* Since none of these studies examined maternal and fetal pairs in PE and FGR, it is uncertain whether common pathways are triggered in both the mother and the fetus.

In this study, we aimed to investigate the metabolomic profile of paired maternal and cord blood plasma in PE and FGR by nuclear magnetic resonance (NMR) spectroscopy, which provides a quantitative and unbiased assessment of lipidomic and metabolomic profiles in a given sample.

## Results

### Study population

Table 1 displays the description of the study population. A total of 222 patients were included in this study. The study groups were similar in terms of maternal age, parity and mode of conception. Chronic hypertension was significantly higher among PE cases, while pregestational body mass index (BMI) was signigifcantly higher in mothers having PE without FGR. A high percentage of smoking women was observed in normotensive FGR with lower pregestational BMI compared to controls. As expected, FGR groups presented higher uterine and umbilical arteries pulsatility index (PI) on Doppler ultrasound assessment and lower fetal middle cerebral artery PI and cerebroplacental ratio, whereas in PE without FGR Doppler parameters were similar to controls. At delivery, gestational age was earlier in PE and FGR with a higher rate of cesarean sections compared to controls.

**Table 1:** Maternal and perinatal characteristics of the study population. Controls are normotensive pregnancies with appropriate growth for gestational age fetuses. Perinatal mortality was defined as stillbirth or neonatal mortality within 28 days of delivery. BMI, body mass index; PI, pulsatility index.

	Controls n = 88	FGR n = 44	PE n = 40	PE + FGR n = 50
**Maternal characteristics**				
Age (years)^μ^	34.4 (31.2–36.8)	34.8 (29.3–39.1)	33.6 (30.2–36.6)	35 (31.6–37.6)
**Ethnicity**^**n**^				
*Caucasian*	51 (58)	33 (75)*	16 (40)	24 (48)
*African*	6 (6.8)	2 (4.5)	3 (7.5)	8 (16)
*Latin*	20 (22.7)	1 (2.3)*	8 (20)	11 (22)
*Asian*	11 (12.5)	8 (18.2)	13 (32.5)*	7 (14)
Pre-gestational BMI (kg/m^2^)^μ^	22.5 (20.6–24.2)	21.6 (19.8–23.3)	25.2 (22.1–28.9)*	23.8 (21.1–26.2)
Chronic hypertension^η^	0 (0)	0 (0)	5 (12.5)*	6 (12)*
Nulliparity^η^	51 (58)	25 (56.8)	25 (62.5)	35 (70)
Assisted reproductive technologies^η^	5 (5.7)	6 (13.6)	4 (10)	5 (10)
Smoking during pregnancy^η^	5 (5.7)	10 (22.7)*	2 (5)	5 (10)
**Feto-placental Doppler**				
Uterine arteries mean PI (z score)^μ^	− 0.27 (− 1.06–0.79)	0.9 (− 0.22–2.76)*	− 0.67 (− 1.69–0.7)	2.55 (1.96–3.24)*
Umbilical artery PI (z score)^μ^	− 0.15 (− 0.53–0.23)	0.23 (− 0.22–1.4)*	− 0.11 (− 0.52–0.13)	0.51 (0.04–1.41)*
Middle cerebral artery PI (z score)^μ^	0.15 (− 0.44–0.89)	− 0.34 (− 1.04–0.11)*	− 0.22 (− 0.8–0.32)	− 0.84 (− 1.63–− 0.11)*
Cerebroplacental ratio (z score)^μ^	− 0.06 (− 0.78–0.86)	− 1.01 (− 2.32–0.06)*	− 0.31 (− 0.96–0.23)	− 1.47 (− 2.42–− 0.65)*
**Perinatal outcomes**				
Gestational age at delivery (weeks)^μ^	39.6 (37.2–40.6)	37.6 (37.1–38.4)*	37.5 (36.9–38.7)*	35.1 (32.9–37.3)*
Preterm deliveries < 37 weeks gestation^η^	18 (20.5)	10 (22.7)	10 (25)	32 (64)*
Cesarean section^η^	25 (28.4)	21 (47.7)*	23 (57.5)*	27 (54)*
Male gender^η^	53 (60.2)	23 (52.3)	15 (37.5)*	29 (58)
Birthweight (g)^μ^	3317 (2823–3610)	2175 (1810–2424)*	2937 (2665–3181)	1804 (1484–2342)*
Birthweight centile^μ^	47 (32–67)	1 (0–2)*	47 (21–72)	1 (0–3)*
APGAR score 5 min < 7^η^	0 (0)	0 (0)	1 (2.5)	2 (4)
Umbilical artery pH^μ^	7.21 (7.16–7.27)	7.24 (7.19–7.3)	7.22 (7.19–7.24)	7.23 (7.12–7.26)
Admission to neonatal intensive care unit^η^	10 (11.4)	12 (27.3)*	8 (20)	29 (58)*
Perinatal mortality^η^	0 (0)	1 (2.3)	0 (0)	2 (4)

^μ^Data are median (interquartile range).

^η^Data are n (%).

*p < 0.05 by Mann Whitney U test, Pearson χ^2^ or Fisher exact tests as appropriate, compared to controls.

### Metabolomics of plasma samples

**Figure 1:**
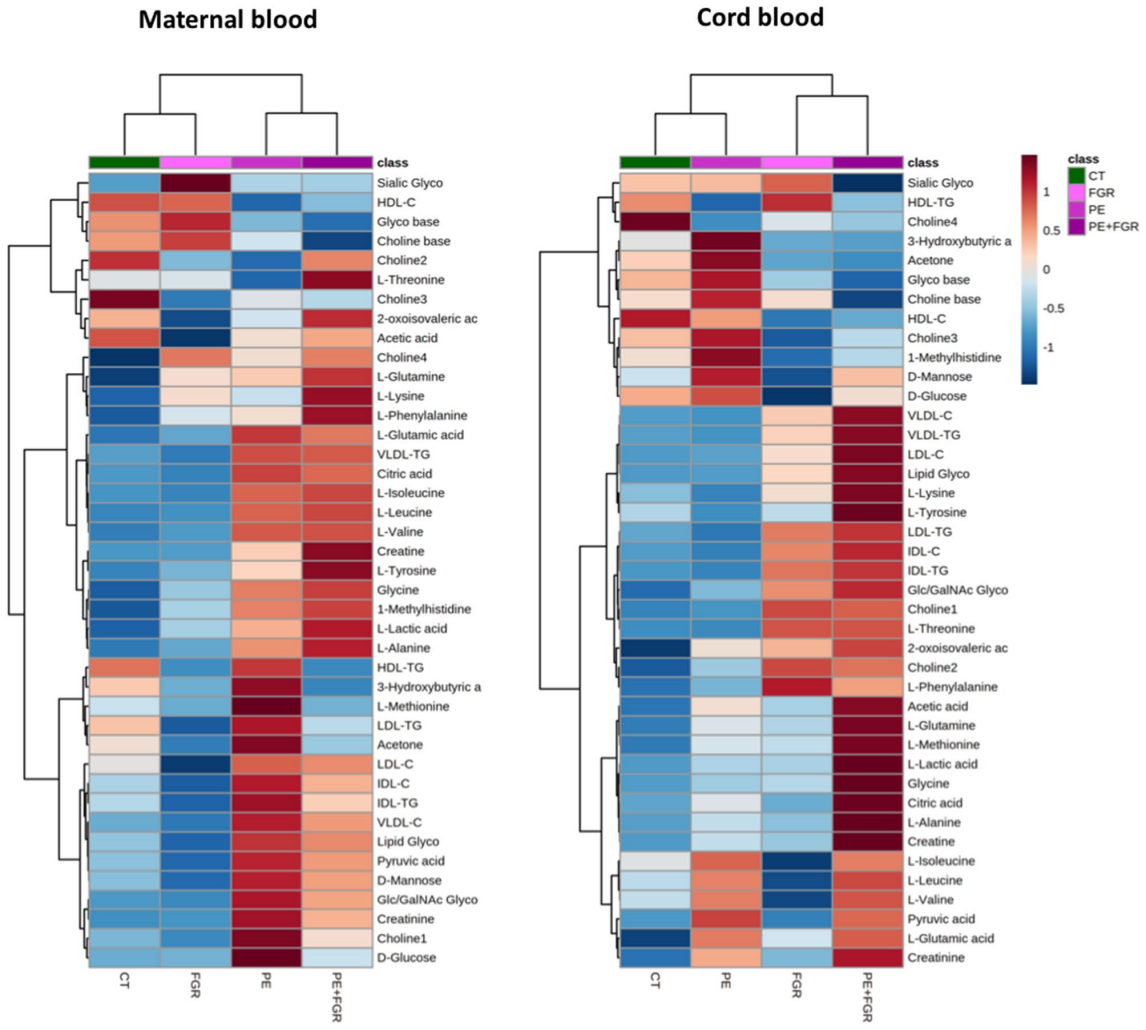
Metabolomic heat maps. Left-side, maternal (controls [n = 88], FGR [n = 44], PE [n = 40], PE + FGR [n = 50]); right-side, cord blood (controls [n = 86], FGR [n = 43], PE [n = 37], PE + FGR [n = 50]) plasma samples. A z-score transformation was performed on the intensity of each metabolite across all the study groups and each group’s mean z-score is displayed in the heatmap. Rows represent metabolites and columns represent samples. Samples and metabolites are clustered by Euclidean distance and Ward linkage. CT, controls; FGR, fetal growth restriction; PE, preeclampsia; VLDL, Very low-density lipoprotein; IDL, Intermediate-density lipoprotein; LDL, Low-density lipoprotein; HDL, High-density lipoprotein; C, cholesterol; TG, triglycerides; Glc/GalNAc, N-acetylglucosamine and N-acetylgalactosamine bonds glycoproteins.

**Figure 2:**
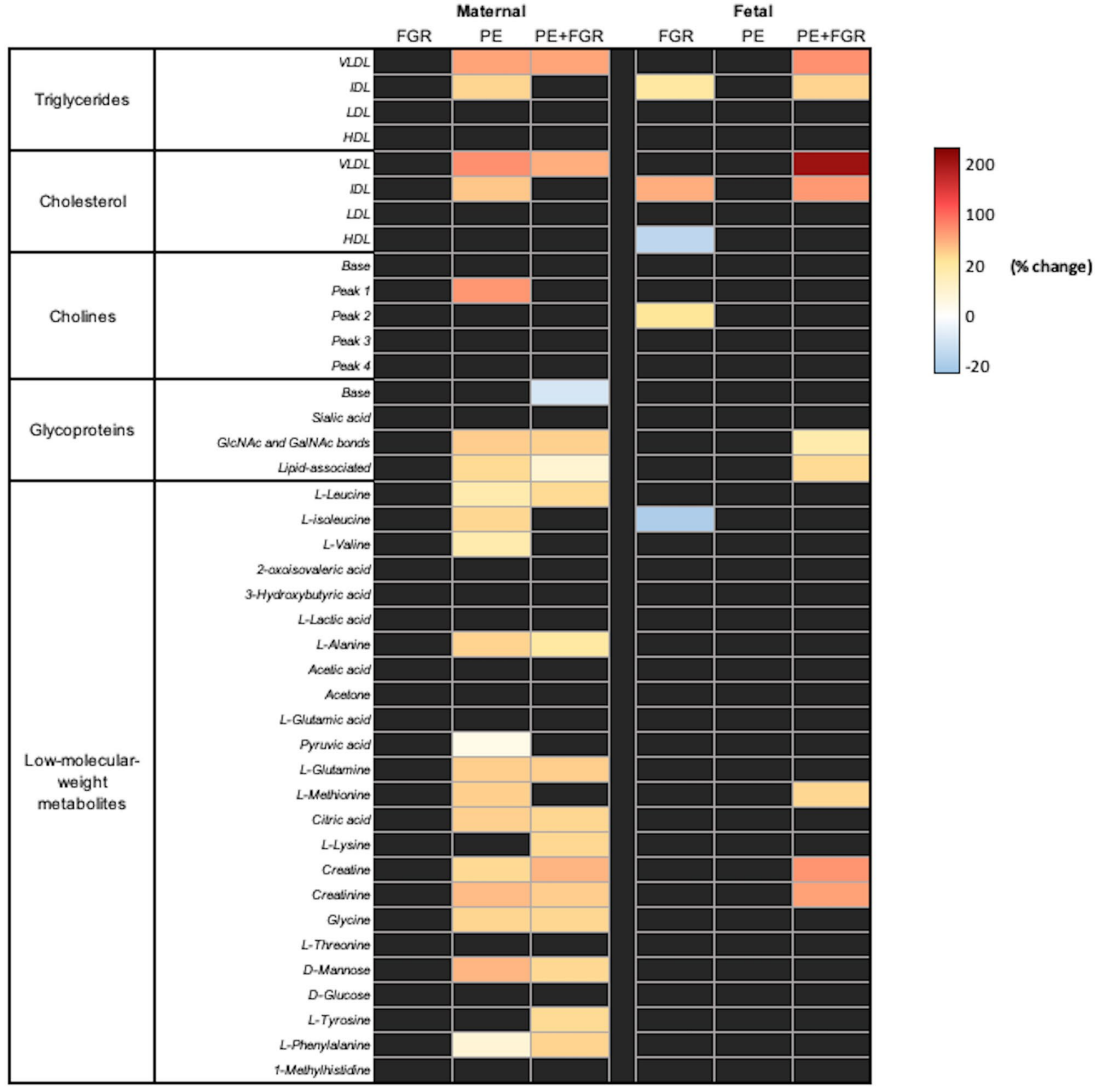
Metabolomic heat maps. Left-side, maternal (controls [n = 88], FGR [n = 44], PE [n = 40], PE + FGR [n = 50]); right-side, cord blood (controls [n = 86], FGR [n = 43], PE [n = 37], PE + FGR [n = 50]) plasma samples. The colored heat map represent the percentage change of the metabolite medians for cases versus controls, following a graded color scale from blue (− 20%) to yellow (+ 20%), to red (+ 200%). Only significant changes are displayed, according to the Mann Whitney U test and confirmed by linear regression adjustment for maternal body mass index, smoking, sample collection date, gestational age at delivery/sampling, route of delivery and fetal gender after 5% false discovery rate correction (Supplementary Table S1). FGR, fetal growth restriction; PE, preeclampsia; VLDL, Very low-density lipoprotein; IDL, Intermediate-density lipoprotein; LDL, Low-density lipoprotein; HDL, High-density lipoprotein; Glc/GalNAc, N-acetylglucosamine and N-acetylgalactosamine bonds.

A first-line analysis of the lipid and low-molecular-weight metabolite profiles indicated more prevalent changes in PE and PE + FGR mothers, and stronger changes in PE + FGR fetuses (Fig. 1). These differences were further analyzed after statistical analysis of the quantitative datasets, including adjustment according to maternal BMI, smoking, sample collection date, gestational age at delivery/sampling, route of delivery and fetal gender and false discovery rate correction compared to controls (Supplementary Table S1), and are summarized in Fig. 2. Overall, normotensive FGR mothers and non-FGR PE fetuses revealed no significant changes in lipids or low-molecular-weight metabolites. The strongest changes detected were associated with VLDL-triglycerides, VLDL-cholesterol and choline compounds, within the 1.5–1.6 fold (50–60%) range in PE and PE + FGR mothers, and increasing to 1.5–2.8 fold (50–180%) in PE + FGR fetuses; whereas changes in low-molecular-weight metabolites were mostly below those ranges in PE mothers, with or without FGR, and to a less extent in PE + FGR fetuses.

### Lipid profiles

**Figure 3:**
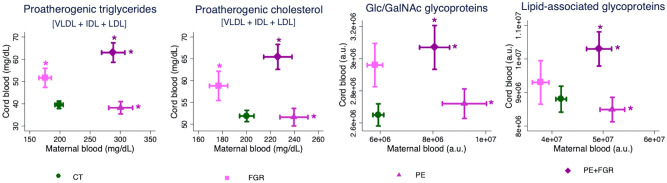
Lipid profiles across the different groups. Proatherogenic triglycerides and cholesterol, and Glc/GalNAc and lipid-associated glycoproteins in plasma samples from maternal blood (X-axis: controls [n = 88], FGR [n = 44], PE [n = 40], PE + FGR [n = 50]) and cord blood (Y-axis: controls [n = 86], FGR [n = 43], PE [n = 37], PE + FGR [n = 50]). The plots show the mean values in each group + /− SEM. * p < 0.05 according to Student’s *t*-test or Mann Whitney U test and confirmed by linear regression (adjusted for maternal body mass index, smoking, sample collection date, gestational age at delivery/sampling, route of delivery and fetal gender) and 5% false discovery rate correction compared to controls (Supplementary Table S1). CT, controls; FGR, fetal growth restriction; Glc/GalNAc, N-acetylglucosamine and N-acetylgalactosamine bonds; PE, preeclampsia.

Mother-fetus pairs were further analyzed after grouping the lipoproteins as pro- and anti-atherogeneic (VLDL + IDL + LDL and HDL, respectively). Thus, consistent profile changes were detected for proatherogeneic triglycerides and cholesterol, and N-acetylglucosamine and N-acetylgalactosamine (Glc/GalNAc) and lipid-associated glycoproteins: all similarly disturbed (increased compared to controls) in FGR fetuses with or without PE, and in PE mothers, with or without FGR (Fig. 3); whereas anti-atherogeneic cholesterol was decreased in cord blood of FGR fetuses from normotensive mothers compared to fetuses from uncomplicated pregnancies. These changes were mirrored by choline-compound increases in PE mothers and FGR fetuses (Fig. 2). Additional observations included a more heterogenous distribution of proatherogeneic triglycerides compared to controls in FGR fetuses with or without PE, and in PE mothers with or without FGR (2–2.5 fold larger IQR of VLDL), similar to Glc/GalNAc glycoproteins in the latter groups (Supplementary Table S1).

### Profiles of low-molecular-weight metabolites

**Figure 4:**
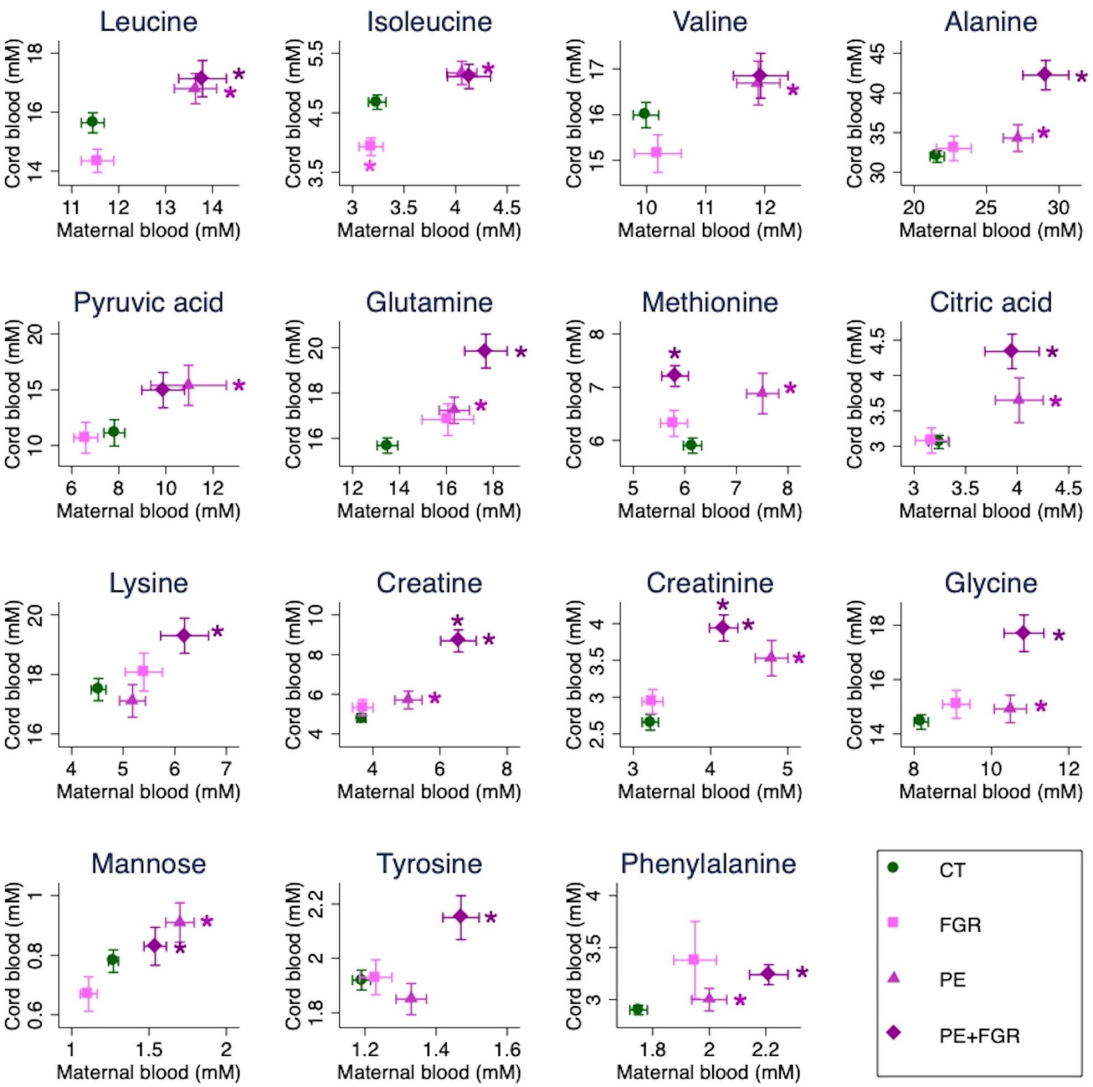
Low-molecular-weight metabolite profiles across the different groups. Plasma samples from maternal (X-axis: controls [n = 88], FGR [n = 44], PE [n = 40], PE + FGR [n = 50]) and cord blood (Y-axis: controls [n = 86], FGR [n = 43], PE [n = 37], PE + FGR [n = 50]) plasma samples. The plots show the mean values in each group + /− SEM. * p < 0.05 according to Student’s *t*-test or Mann Whitney U test and confirmed by linear regression (adjusted for maternal body mass index, smoking, sample collection date, gestational age at delivery/sampling, route of delivery and fetal gender) and 5% false discovery rate correction compared to controls (Supplementary Table S1). CT, controls; FGR, fetal growth restriction; PE, preeclampsia.

Significant changes were detected in a total of 15 low-molecular-weight metabolites (Fig. 2), and further analysed in mother-fetus pairs (Fig. 4). While some of these changes were common to PE + FGR mothers and fetuses (increasedcreatine and creatinine compared to control mothers and fetuses), most were specific to PE mothers with or without FGR (increased leucine, mannose, citrate, alanine, glutamine, glycine and phenylalanine compared to controls). Increased methionine was observed in PE + FGR fetuses and PE mothers (but not PE + FGR mothers) compared to controls. The only metabolic changes consistent with decreased profile levels were detected for isoleucine in normotensive FGR fetuses (and similar tendencies for leucine and valine; while increasing in PE mothers).

## Discussion

This study explores for the first time the profile of maternal and fetal metabolomics in the different phenotypes of PE and FGR. Lipids were altered in accordance with the clinical presentation of these disorders, i.e. in PE mothers and FGR fetuses. Low-molecular-weight metabolites were disturbed mainly in preeclamptic mothers and growth restricted fetuses in the presence of PE, while few significant differences were found in normotensive FGR fetuses.

In the present study, normotensive FGR mothers exhibited no significant differences in lipid or low-molecular-weight metabolite profiles compared to controls. However, a trend towards lower concentrations of triglycerides and remnant cholesterol (VLDL and IDL), and lipid-associated glycoproteins was observed, in line with the existing literature^14,16,18,19^. The main findings in cord blood plasma samples were elevated IDL triglycerides and cholesterol, which is also consistent with the literature^14,15^. Surprisingly, this study shows for the first time that growth restricted fetuses from normotensive mothers have little differences in low-molecular-weight metabolites compared to uncomplicated pregnancies. These differences are consistent with undernutrition, manifested by lower levels of the essential amino acid isoleucine; and similar trends for the additional branched chain amino acids, leucine and valine. Previous studies included heterogenous populations of growth restricted fetuses with or without PE^15,16^.

On the other hand, our observation of elevated maternal lipids in PE compared to controls (namely, proatherogenic triglycerydes and cholesterol, choline compounds, and glycoproteins) is not unanticipated^20,21^. It is well established that PE is associated with disturbed maternal cholesterol metabolism and related oxidative stress^22,23^. In terms of low-molecular-weight metabolites, the majority were deregulated in PE mothers. Some of these metabolites have been reported altered from the first trimester in women who later developed PE: branched-chain amino acids (leucine, isoleucine and valine)^24^, implicated in cell signaling and related to insulin resistance and risk of developing diabetes^25^; methionine^26^, a key player in angiogenesis and antioxidation^27^; alanine and phenylalanine^28^, important amino acids in the biosynthesis of proteins; and mannose, an essential sugar for glycoprotein synthesis, consistent with their increase in this phenotype. Previous studies reported varying results while investigating the metabolome signature of PE, apparently due to differences in case selection, gestational age at assessment, platforms and protocols^8^. In fact, all of these studies included a mixed population of PE cases with or without FGR, and did not explore the fetal metabolomic profile in PE without FGR. Thus, our data show for the first time no significant differences in any of the studied metabolites in these fetuses.

PE + FGR mothers also presented a disturbed metabolomic profile compared to controls, including higher proatherogenic triglycerides and cholesterol, glycoproteins and significant increases in most low-molecular-weight metabolites, similar to the maternal profile in PE without FGR. On the other hand, cord blood metabolomics showed the impact of fetal growth pattern in pregnancies complicated by PE on the fetal lipid profile. Indeed, PE + FGR fetuses showed exceedingly high and heterogeneous concentrations of proatherogenic triglycerides and cholesterol, as well as increased choline compounds and glycoproteins; whereas no differences were observed in PE without FGR compared to fetuses from uncomplicated pregnancies. Moreover, creatine and creatinine were increased in both PE + FGR mothers and fetuses. Previous studies showed less marked differences in PE + FGR fetuses, likely due to a smaller number of cases^19,29^. Curiously, the decreases in antiatherogenic cholesterol (HDL) and isoleucine (essential anino acid) detected in normotensive FGR fetuses were not observed in PE + FGR fetuses, suggesting no undernutrition of the fetus in the latter.

The placenta is the keyplayer in the transfer of nutrients and oxygen from mother to fetus^30^. A physiological maternal adaptation during pregnancy is essential to supply cholesterol and triglycerides to the growing fetus^31^. Placental insufficiency may therefore generate specific maternal and fetal responses that might be distinct in the different phenotypes of PE and FGR. The trend towards low concentrations of circulating maternal lipids in normotensive FGR might reflect a status of undernutrition in these mothers that facilitates the development of FGR^32^; or it could be due to an increased uptake by a defective placenta, since maternal lipoproteins scarcely cross the placental barrier^33^. PE mothers, on the other hand, exhibited higher lipids, glycoproteins and disturbed metabolomic profile, which suggests a status of systemic inflammation, endothelial dysfunction and multi-organ impairment^34,35^. Namely, Glc/GalNAc-glycoproteins have been associated with chronic inflammation^36^. On the flip side, fetal lipoproteins, mostly synthesized in the fetal liver, are increased in growth restricted fetuses, probably as a response to undernutrition and hypoxia. The novel insights of the current study reveal divergent fetal profiles of low-molecular-weight metabolites in FGR, with or without PE: more disturbed in PE + FGR versus scarce differences in normotensive FGR. Matched maternal and fetal metabolomic responses in PE + FGR are thus relevant. The high concentrations and similar distribution of low-molecular-weight metabolites in maternal and fetal plasma in this group may arise from their passive transfer through the placenta due to a defective placental barrier associated with FGR. Alternatively or in parallel, toxic compounds in maternal circulation associated with PE, such as reactive oxygen species^37^, may cross the placenta and affect the mother and the developing fetus in a similar fashion. This interplay between maternal and fetal compartments is complex, with different patterns of placental transport and metabolism of amino acids, sugars and other metabolites^38^, which merit further attention beyond the scope of the current study.

The strengths of this study include its prospective design, the inclusion of a large number of well-characterized pregnancies and the collection of paired maternal and fetal plasma samples at the time of delivery. The diagnosis was confirmed in all participants and uncertain cases were excluded. From a methodological standpoint, one of the main advantages of NMR is its reproducibility, which makes this technique suitable for fingerprinting analysis, such as the study reported herein. Moreover, since the peak area of a compound in NMR spectroscopy can be directly related to its relative or absolute concentration, this makes the quantification of different metabolites very precise^39^. Yet, we acknowledge some limitations. First, matching for gestational age at sampling and the timing of cord clamping was not possible due to the higher rate of medically-indicated earlier delivery in PE and FGR. Second, the current study was essentially composed of late-onset cases, denoting the most prevalent form for both PE and FGR. Even though, we also included 18 preterm uncomplicated pregnancies (normotensive without FGR, fetal infection or any other anomalies) in the analysis. Importantly, since the maternal metabolic profile changes during pregnancy^40^, differences in gestational age at delivery/sampling were included as potential confounders in the statistical analysis. Third, the study undertook 3 years and therefore storage time was different between early and late-collected samples. To adjust for this difference, sample collection date was included in the regression analysis. Fourth, a fasting status preceding maternal blood collection was not assured due to the study design that aimed to collect maternal and fetal plasma samples within a narrow interval at the time of delivery. This might explain partially the higher heterogeneity of triglycerides in PE mothers in this study. While there is a marginal effect of fasting on LDL-cholesterol and VLDL-triglycerides (leading to slightly elevated and reduced levels, respectively), they are usually not significant in clinical practice^41^. Although our findings are consistent with the literature, the low-molecular-weight metabolites analysis would benefit from more sensitive techniques (e.g. liquid chromatography mass spectrometry), to explore a wider range of metabolites and ascertain pathway-specific changes; particularly in PE mothers. This warrants further investigation.

The present study reinforces the evidence towards a disturbed lipid metabolism in PE mothers and FGR fetuses, manifested by elevated VLDL, IDL and depleted HDL, i.e. a pro-atherogenic lipoprotein profile. Such hyperlipidemia may contribute to the increased cardiovascular risk in the affected subjects through their life course^3,4,42^. Additionally, several metabolite disturbances in PE mothers mirror lifestyle factors, which could help tailoring dietetic advice in high-risk women. Of interest, fetal metabolomics in growth restricted fetuses is impacted by the presence of PE. Normotensive FGR is associated with signs of fetal undernutrition that are not present in PE + FGR. Further research is warranted to clarify maternal and fetal adaptations to PE and FGR and their short and long term consequences. Moreover, investigating the placental role in the transfer of low-molecular-weight metabolites and the interplay between the maternal and fetal compartments would be of particular relevance.

## Methods

### Study population

This was a prospective observational study including singleton pregnancies with the diagnosis of PE and FGR who attended the Department of Maternal–Fetal Medicine of BCNatal (Barcelona, Spain) between January 2015 and December 2017. PE was defined as high blood pressure (systolic blood pressure ≥ 140 mmHg and/or diastolic blood pressure ≥ 90 mmHg on two occasions, at least four hours apart), developed after 20 weeks of gestation, with proteinuria (≥ 300 mg/24 h) or protein/creatinine ratio ≥ 0.3^1,43^. FGR was defined as estimated fetal weight (EFW) and birthweight below the 10th centile associated with either abnormal cerebroplacental ratio (< 5th centile) or abnormal uterine artery pulsatility index (> 95th centile), or birthweight below the 3rd centile^44^. The EFW and birthweight centiles were assigned according to local standards^45^. Uncomplicated pregnancies with normotensive mothers and appropriate growth for gestational age fetuses -defined as EFW and birthweight above the 10th centile- were randomly selected from our general population and included as controls. In all pregnancies, gestational age was calculated based on the crown-rump length at first trimester ultrasound^46^. Pregnancies with pregestational diabetes, familial hypercholesterolemia, chromosomal/structural anomalies or intrauterine infection were excluded. Patient selection and sampling procedures were performed in accordance with the Declaration of Helsinki and applicable local regulatory requirements after approval from the Ethics Committee of the Hospital Clinic of Barcelona (HCB/2016/0253). Participating patients provided their written informed consent.

### Data collection and study protocol

The following data were recorded upon enrollment: maternal age, ethnicity, BMI, parity, known chronic disease, mode of conception, smoking status, and obstetric history. Feto-placental Doppler parameters were obtained within the 2 weeks preceding the delivery including uterine, umbilical and fetal middle cerebral arteries pulsatility indices and the calculation of the cerebroplacental ratio. These values were normalized into z-scores accordingly^47–49^. At delivery, gestational age, birthweight, birthweight centile, Apgar scores, umbilical artery pH, admissions to the neonatal intensive care unit and perinatal mortality were recorded.

### Sample collection and storage

Maternal blood samples were drawn within 2 h after delivery (at least eight hours after their last meal). Fetal umbilical cord blood samples were obtained from the clamped umbilical cord immediately after delivery. All blood samples were collected in EDTA-treated tubes. Plasma was separated by centrifugation at 1500 g for 10 min at 4 °C, identified anonymously (based on the diagnosis, not collection date), and stored at − 80 °C until further use.

### NMR data acquisition

A general metabolomic profile (small molecules, amino acids, organic acids, lipoproteins and glycoproteins) were measured by using Nuclear Magnetic Resonance (1H-NMR) spectroscopy for metabolome quantification. Plasma samples were thawed overnight and processed to obtain NMR profiles according to the Bruker-specific metabolomics protocol^50^. Aliquots from each sample (300 μL) were mixed with sodium phosphate buffer (300 μL) for immediate analysis. High-resolution ^1^H-NMR spectroscopy data were acquired at the Andalusian Center for Nanomedicine and Biotechnology (BIONAND, Malaga), on a Bruker 600 MHz Spectrometer (Bruker Biospin, Rheinstetten, Germany) equipped with an Avance III console and a TCI CryoProbe Prodigy: 1D Nuclear Overhauser Effect SpectroscopY (NOESY), Carr-Purcell-Meiboom-Gill (CPMG), and 2D j-resolved spectroscopy (JRES), all with pre-saturation of the residual water peak, to characterize small molecules such as amino acids and sugars; and 1D Diffusion (Diff), to detect larger molecules such as lipoproteins, choline compounds and glycoproteins^51–55^. All the sequences were run at 37 °C in quantitative conditions (systematic pre-calibration of radiofrequency pulses and sample temperature, and same receiver gain adjustment). CPMG and Diff data were preprocessed at the NMR console (TopSpin 3.2, Bruker Biospin, Rheinstetten, Germany) for basic corrections, such as phase correction and exponential line broadening (0.5 Hz for CPMG; 1.0 Hz for Diff). On initial metabolomic analysis, readers were blinded to patient status.

### Lipid profiling

Lipid profiling was based on the Liposcale test, which quantifies the main classes of lipoproteins [very low-density lipoprotein (VLDL), low-density lipoprotein (LDL), intermediate-density lipoprotein (IDL) and high-density lipoprotein (HDL)] for cholesterol and triglycerides^56,57^. The signals from choline compounds and glycoproteins were resolved by spectral deconvolution into four and three Lorentzian analytical functions, respectively. The three glycoprotein functions were classified from highest to lowest chemical shift in (1) Sialic acid, (2) Glc/GalNAc, and (3) Lipid-associated, according to previous published methodology^36^. For each of these functions, the total area (proportional to concentration) was determined. The method has been previously validated for plasma samples of adult and cord blood, over a wide concentration range of lipoproteins, thus providing quantitative measurements for triglycerides and cholesterol estimations^14^.

### Low-molecular-weight metabolite profiling

CPMG data were used for a targeted profiling of low-molecular-weight metabolites, based on a new, fully automated version of the Dolphin software package (version 0.2.0 available from https://github.com/danielcanueto/rDolphin)^58,59^, as done before for adult and cord blood samples^14^. This software uses the region of interest (ROI) concept^60^ to allow for flexible and time-affordable automatic profiling of 1D 1H-NMR spectra. In addition, the package implements an approach based on the estimation of the baseline and the signal parameter values (chemical shift, intensity, half bandwidth, j-coupling), which maximize the quality in the lineshape fitting of the signals in the analysed ROI, despite the differences between cord blood and maternal blood. The absolute quantification of low-molecular-weight metabolites (mM) was based on a frequency-domain fitting analysis of specific spectral regions. Since the spectral area is equivalent to the molecular abundance, individual signal areas were converted into molar concentrations through a normalization factor (the number of protons generating each signal). The used full automated version of the software outputs several indicators of quality and reliability: fitting error, ratio between quantified signal area and total spectrum region area, expected chemical shift, expected half bandwidth, expected intensity. The last three indicators are novel information sources enabled by Machine learning based prediction of these signal parameters according to information extracted from signals correlated to the one of interest. The analysis of these parameters facilitates the review of the results and the selection of those that should be discarded. After removing unreliable signals, 24 metabolites were analyzed including amino acids and sugars: leucine, isoleucine, valine, 2-oxoisovaleric acid, 3-hydroxybutyrate, lactate, alanine, acetate, acetone, glutamate, pyruvate, glutamine, methionine, citrate, lysine, creatine, creatinine, glycine, threonine, mannose, glucose, tyrosine and 1-methylhistidine. The absolute quantification of low-molecular-weight metabolites (mM) was based on a frequency-domain fitting analysis of specific spectral regions. Since the spectral area is equivalent to the molecular abundance, individual signal areas were converted into molar concentrations through a normalization factor (the number of protons generating each signal). Signal annotation was based on templates prepared in previous studies with the help of available databases^61^ and bibliography. Validation of metabolite identification was assisted by STOCSY^62^ and JRES data.

### Statistical analysis

Clinical and metabolomic data were analyzed with the statistical software STATA 14.2 (StataCorp LLC, Texas, USA). Metaboanalyst 4.0 (http://www.metaboanalyst.ca/) was also used in the analysis of metabolomic data. Results are expressed as median (interquartile range) or number (percentage) as appropriate. Statistical analysis comprised the comparison of each group of the cases vs. the controls. For metabolomic data, distributions were examined for normality using the Kolmogorov–Smirnov test. When the distribution was normal, unpaired t-tests was used. Otherwise, the Mann–Whitney U-test was used. The Benjamini–Hochberg method was used to adjust p values for multiple testing with consideration of 5% false discovery rate. In addition, results were adjusted by linear regression for potential confounding factors including pregestational BMI, smoking, sample collection date (since differences in storage periods of the samples included might affect their metabolomic profile), gestational age at delivery/sampling, route of delivery and fetal gender. A heat map was generated to illustrate mean intensity of each metabolite across the study groups (z-scores), and the percentage of fold change of each metabolite for each group of the cases vs. the controls. There were no missing data in maternal blood samples, whereas 6 cord blood samples had insufficient volume for NMR analysis (metabolomic data not available). Since this missing data represents only 1.3% of the whole dataset we considered it irrelevant for the statistical analyses performed. All reported *p* values are 2 sided. Differences were considered significant when p < 0.05.

## Supplementary Information

Supplementary Information 1.

Supplementary Information 2.

## Data Availability

The metabolomics quantification data reported in this study are available as supplementary information.
