# Supplementary material for: Paired maternal and fetal metabolomics reveal a differential fingerprint in preeclampsia versus fetal growth restriction
Source: Sci Rep. 2021 Jul 13;11:14422. doi: 10.1038/s41598-021-93936-9 (PMC8277896; doi:10.1038/s41598-021-93936-9)
Supplement: Supplementary file 1 — Supplementary Information 1. [file 41598_2021_93936_MOESM1_ESM.docx]

**Paired maternal and fetal metabolomics reveal a differential fingerprint in preeclampsia versus fetal growth restriction**

Lina Youssef MD PhD^1^, Rui V Simões PhD^1,‡*^, Jezid Miranda MD PhD^1^, García-Martín ML^2^, Cristina Paules MD PhD^1^, Francesca Crovetto MD PhD^1^, Nuria Amigó PhD^3^, Nicolau Cañellas PhD^4,5^, Eduard Gratacos MD PhD^1,6^, Fatima Crispi MD PhD^1,6^.

^1^BCNatal | Fetal Medicine Research Center (Hospital Clínic and Hospital Sant Joan de Déu), Institut d'Investigacions Biomèdiques August Pi i Sunyer (IDIBAPS), University of Barcelona, Barcelona, Spain.

^‡^Current address: Champalimaud Research, Champalimaud Centre for the Unknown, Lisbon, Portugal.

^2^BIONAND, Andalusian Centre for Nanomedicine and Biotechnology (Junta de Andalucía- Universidad de Málaga), Málaga, Spain.

^3^Biosfer Teslab, Reus, Spain. Department of Basic Medical Sciences, University Rovira I Virgili, CIBERDEM, Reus, Spain

^4^Universidad Rovira i Virgili, DEEEiA, IISPV, Tarragona, Spain.

^5^CIBERDEM, Spanish Biomedical Research Centre in Diabetes and Associated Metabolic Disorders, Madrid, Spain.

^6^Centre for Biomedical Research on Rare Diseases (CIBER-ER), Madrid, Spain.

*Corresponding author: Rui Simoes, PhD, address: Champalimaud Research, Champalimaud Centre for the Unknown, Av Brasília, Av Brasília, 1400-038 Lisbon, Portugal; e-mail: [rui.simoes@research.fchampalimaud.org](https://webmail.clinic.cat/owa/redir.aspx?C=Br1jqC5oM-hbLj1I8RXMmFqdO0blah-tHs2dYKyo4AkFRBhn18zYCA..&URL=mailto%3arui.simoes%40research.fchampalimaud.org)

**Supplementary information**

**Supplementary Table S1.** Concentrations of lipoproteins, cholines, glycoproteins and low-molecular-weight metabolites in maternal and cord blood plasma samples across the study groups.

|  | | ***Maternal blood*** | | | | ***Cord blood*** | | | |
| --- | --- | --- | --- | --- | --- | --- | --- | --- | --- |
|  | **Controls**  n=88 | | **FGR**  n=44 | **PE**  n=40 | **PE+FGR**  n=50 | **Controls**  n=86 | **FGR**  n=43 | **PE**  n=37 | **PE+FGR**  n=50 |
| *Lipids* |  | |  |  |  |  |  |  |  |
| Triglycerides (mg/dL) |  | |  |  |  |  |  |  |  |
| VLDL | 129.3  (94.3 – 170) | | 110.4  (83.3 – 153.6) | 197.7  (129.7 – 310)*†λ | 196.7  (133.5 – 287.9)*†λ | 25.2  (21.9 – 37.2) | 32.4  (23.2 – 45.2)*† | 23.6  (21.4 – 31.5) | 41.1  (29.4 – 58.9)*†λ |
| IDL | 23.9  (20.7 – 29.8) | | 23  (19.1 – 26.7)* | 29.8  (24.9 – 33.4)*†λ | 26.5  (21.2 – 31.1) | 5.07  (3.87 – 6.3) | 6.01  (4.95 – 7.9)*†λ | 4.61  (3.67 – 5.94) | 6.38  (5.3 – 8.99)*†λ |
| LDL | 33.5  (28.1 – 43.9) | | 30.2  (24.3 – 38.3)* | 39.3  (29 – 47.5) | 32.9  (28 – 40) | 3.29  (2.5 – 5.2) | 4.42  (3.26 – 6.98)* | 3.09  (1.64 – 5.57) | 5.15  (3.63 – 7.18)*† |
| HDL | 30.1  (25.6 – 34.7) | | 26.3  (20.3 – 31.9)* | 32.3  (27.7 – 35.6) | 26.3  (20.5 – 33.9)*† | 8.08  (7.23 – 8.76) | 7.8  (7.28 – 8.57) | 7.54  (6.83 – 8.58) | 8.26  (6.81 – 9.21) |
| Cholesterol (mg/dL) |  | |  |  |  |  |  |  |  |
| VLDL | 34.8  (24.5 – 45.5) | | 27.9  (21.9 – 37.9) | 57  (36.5 – 72)*†λ | 51  (33.1 – 64.6)*†λ | 2.89  (1.23 – 6.88) | 4.56  (2.21 – 10.06)*† | 1.92  (0.84 – 5.94) | 8.02  (4.01 – 15.13)*†λ |
| IDL | 25.7  (21.8 – 33.4) | | 23.2  (19.5 – 29.1)* | 34.1  (27.7 – 38.2)*†λ | 30.3  (23.8 – 37.9)* | 3.25  (2.02 – 4.3) | 4.77  (3.1 – 6.3)*†λ | 2.45  (1.73 – 4.86) | 5.16  (3.63 – 7.17)*†λ |
| LDL | 127.3  (104.7 – 159) | | 121.9  (99.2 – 141.9)* | 143  (116.3 – 177.7) | 128.9  (107.4 – 154.4) | 42.6  (38.8 – 47.3) | 44.2  (37.9 – 49.3) | 44.7  (38.4 – 50.3) | 44.4  (39.2 – 58.5)*† |
| HDL | 48.7  (38 – 60.7) | | 46.9  (38.8 – 62.8) | 39.4  (30.4 – 49.5)*† | 42.1  (29.1 – 55.8) | 33.2  (29.3 – 39.3) | 28.9  (23.3 – 37.9)*λ | 33  (28.9 – 36.7) | 29.4  (24.3 – 34.3)λ |
| *Cholines (a.u.)* |  | |  |  |  |  |  |  |  |
| Base | 1.41  (1.29 – 1.58) × 10^6^ | | 1.47  (1.4 – 1.58) × 10^6^ | 1.39  (1.17 – 1.64) × 10^6^ | 1.42  (1.13 – 1.57) × 10^6^λ | 1.59  (1.49 – 1.73) × 10^6^ | 1.6  (1.46 – 1.8) × 10^6^ | 1.62  (1.5 – 1.78) × 10^6^ | 1.45  (1.32 – 1.72) × 10^6^ |
| Peak 1 | 2.24  (1.56 – 3.11) × 10^6^ | | 2.21  (1.47 – 3.01) × 10^6^ | 3.59  (2.1 – 4.99) × 10^6^*†λ | 2.65  (1.84 – 3.36) × 10^6^ | 0.45  (0.27 – 0.62) × 10^6^ | 0.44  (0.33 – 0.73) × 10^6^* | 0.37  (0.24 – 0.56) × 10^6^ | 0.5  (0.28 – 0.98) × 10^6^*† |
| Peak 2 | 2.13  (1.21 – 3.21) × 10^6^ | | 1.76  (0.99 – 2.61) × 10^6^ | 1.47  (0.75 – 2.71) × 10^6^* | 1.92  (1.18 – 3.35) × 10^6^ | 0.17  (0.09 – 0.29) × 10^6^ | 0.41  (0.18 – 0.82) × 10^6^*†λ | 0.2  (0.14 – 0.56) × 10^6^ | 0.33  (0.14 – 0.76) × 10^6^*† |
| Peak 3 | 5.65  (4.74 – 7.58) × 10^6^ | | 5.37  (3.94 – 6.76) × 10^6^ | 5.86  (4.56 – 7.24) × 10^6^ | 5.58  (4.16 – 6.93) × 10^6^ | 4.45  (3.33 – 5.41) × 10^6^ | 3.68  (1.92 – 4.6) × 10^6^ | 5.01  (3.52 – 5.56) × 10^6^ | 3.92  (2.73 – 4.78) × 10^6^ |
| Peak 4 | 5.06  (3.74 – 6.1) × 10^6^ | | 5.84  (3.79 – 6.95) × 10^6^ | 5.3  (4.07 – 6.95) × 10^6^ | 5.8  (4.25 – 7.27) × 10^6^ | 1.73  (0.9 – 3.29) × 10^6^ | 1.48  (0.81 – 2.63) × 10^6^ | 1.23  (0.77 – 2.1) × 10^6^ | 1.46  (0.68 – 3.09) × 10^6^ |
| *Glycoproteins (a.u.)* |  | |  |  |  |  |  |  |  |
| Base | 17.3  (15.6 – 18.9) × 10^6^ | | 17.7  (17.1 – 18.6) × 10^6^ | 16.9  (15.5 – 18.6) × 10^6^ | 15.9  (15.1 – 18.2) × 10^6^*†λ | 15  (14.1 – 16.1) × 10^6^ | 14.4  (13.3 – 15.3) × 10^6^ | 15.4  (14.4 – 16.7) × 10^6^ | 13.8  (12.8 – 15.3) × 10^6^ |
| Sialic acid | 0.6  (0.45 – 0.83) × 10^6^ | | 0.7  (0.6 – 0.95) × 10^6^* | 0.55  (0.32 – 0.84) × 10^6^ | 0.64  (0.39 – 0.93) × 10^6^ | 0.81  (0.67 – 0.89) × 10^6^ | 0.79  (0.59 – 0.99) × 10^6^ | 0.81  (0.67 – 0.94) × 10^6^ | 0.75  (0.59 – 0.89) × 10^6^ |
| Glc/GalNAc | 5.57  (4.62 – 6.71) × 10^6^ | | 5.38  (4.45 – 6.84) × 10^6^ | 7.22  (6.3 – 11) × 10^6^*†λ | 7.07  (5.59 – 8.18) × 10^6^*†λ | 2.5  (2.3 – 2.91) × 10^6^ | 2.69  (2.41 – 3.34) × 10^6^* | 2.66  (2.46 – 2.98) × 10^6^ | 2.91  (2.2 – 3.72) × 10^6^*†λ |
| Lipid-associated | 41.6  (36.1 – 48.2) × 10^6^ | | 37.6  (32.2 – 42.6) × 10^6^* | 50.6  (39.7 – 61.9) × 10^6^*†λ | 45.4  (38.2 – 56) × 10^6^*† | 8.13  (6.86 – 9.5) × 10^6^ | 8.5  (7.5 – 10) × 10^6^ | 8.25  (6.87 – 9.82) × 10^6^ | 9.86  (7.79 – 12.3) × 10^6^*†λ |
| *Low-molecular-weight metabolites (mM)* |  | |  |  |  |  |  |  |  |
| L-Leucine | 11.1  (9.4 – 12.7) | | 11.4  (10.1 – 12.9) | 12.9  (11.5 – 15)*†λ | 13.5  (11 – 15.3)*†λ | 15.6  (13.6 – 17.4) | 14.6  (12.4 – 16.1)λ | 16.4  (15.1 – 18.7) | 16.1  (14.1 – 18.9)*† |
| L-Isoleucine | 3.1  (2.59 – 3.7) | | 3.19  (2.62 – 3.64) | 3.83  (3.61 – 4.47)*†λ | 3.84  (3.17 – 4.73)*† | 4.63  (3.92 – 5.45) | 3.88  (3.08 – 4.57)*†λ | 5.28  (4.22 – 5.71) | 4.88  (4.13 – 5.9)* |
| L-Valine | 9.98  (8.31 – 11.3) | | 10.3  (8.77 – 11.2) | 11.6  (9.96 – 13.5)*†λ | 11.8  (9.55 – 14.3)*† | 15.6  (14 – 17.9) | 14.7  (13.4 – 16.5) | 16.4  (14.6 – 18) | 16.8  (13.7 – 19) |
| 2-Oxoisovaleric acid | 0.82  (0.59 – 0.99) | | 0.63  (0.51 – 0.82)* | 0.77  (0.56 – 0.99) | 0.81  (0.61 – 1.14) | 1.01  (0.77 – 1.2) | 1.04  (0.86 – 1.55) | 1.01  (0.78 – 1.3) | 1.09  (0.75 – 1.57)*† |
| 3-Hydroxybutyric acid | 15.9  (7.7 – 33.2) | | 9.7  (5.9 – 23.9)* | 17  (6.71 – 49.1) | 7.67  (5.36 – 16.1)*† | 10.1  (4.78 – 17.8) | 7.45  (4.02 – 16.1) | 12.6  (5.92 – 32.4)λ | 5.49  (3.29 – 18.3) |
| L-Lactic acid | 322.3  (238.9 – 435.3) | | 314.4  (244.8 – 440.4) | 325.3  (242 – 441.4) | 334.3  (270 – 477.5) | 411.1  (309.8 – 510.1) | 397.9  (331.4 – 524.2) | 383  (309 – 537.8) | 503.2  (362.8 – 640.4)*† |
| L-Alanine | 21.2  (17.9 – 24.7) | | 20.7  (17.9 – 26.5) | 26.7  (21 – 32.1)*†λ | 25.1  (21.3 – 34)*†λ | 30.3  (26.9 – 37.1) | 29.2  (25.4 – 38.5) | 31.1  (27.9 – 38.2) | 39.1  (33.9 – 48.9)*† |
| Acetic acid | 2.18  (1.63 – 2.95) | | 2.03  (1.48 – 2.78) | 2.5  (1.94 – 3.28) | 2.64  (1.91 – 3.26) | 2.26  (1.68 – 2.66) | 2.38  (1.84 – 3.05) | 2.64  (2.27 – 2.99) | 2.69  (2.05 – 3.32)* |
| Acetone | 7.45  (4.88 – 11.3) | | 5.3  (3.93 – 9.03) | 10  (6.55 – 18) | 6.91  (4.08 – 13.8) | 5.54  (3.49 – 10.4) | 5.18  (2.67 – 8.47) | 6.25  (4.02 – 15.8) | 3.41  (2.03 – 8.32)* |
| L-Glutamic acid | 1.55  (1.24 – 1.96) | | 1.69  (1.16 – 2.12) | 1.78  (1.5 – 2.27)*† | 1.87  (1.43 – 2.26)*† | 2.94  (2.23 – 3.66) | 3.39  (2.63 – 4.07) | 3.31  (2.62 – 5.14) | 3.74  (2.94 – 4.86)*† |
| Pyruvic acid | 7.26  (4.87 – 9.25) | | 6.09  (4.23 – 7.85) | 7.59  (5.9 – 12.2)*†λ | 8.8  (6.21 – 10.7) | 7.81  (5.13 – 12.5) | 8.25  (5.4 – 12) | 8.91  (5.98 – 17.08) | 12.74  (6.54 – 19.3)* |
| L-Glutamine | 12.8  (11.2 – 14.1) | | 14.1  (12.2 – 17.1)* | 16.5  (13.2 – 19.1)*†λ | 16.5  (12.9 – 21.7)*†λ | 15.6  (13.7 – 17.6) | 15.8  (13.7 – 18.9) | 17.1  (14.9 – 19.7) | 19  (15.7 – 23.4)*† |
| L-Methionine | 6.05  (5.05 – 6.92) | | 5.64  (4.65 – 6.61) | 7.74  (6.14 – 8.58)*†λ | 5.5  (4.42 – 7.05) | 5.78  (4.88 – 6.99) | 6.03  (5.05 – 7.42) | 6.62  (5.67 – 8.17) | 7.14  (6.12 – 8.06)*†λ |
| Citric acid | 3.1  (2.55 – 3.85) | | 3.11  (2.47 – 3.55) | 3.95  (3.13 – 4.99)*†λ | 3.84  (2.51 – 5.28)*†λ | 2.92  (2.47 – 3.53) | 2.99  (2.3 – 3.46) | 3.41  (2.21 – 4.06) | 4.25  (3.02 – 5.41)*† |
| L-Lysine | 4.42  (3.44 – 5.55) | | 4.95  (4.08 – 6.45)* | 4.66  (4.04 – 6.31)*† | 5.45  (4.32 – 7.3)*†λ | 17.3  (14.8 – 19.5) | 17.1  (15.7 – 20.7) | 17.6  (15.5 – 18.9) | 19.3  (16.7 – 21.8)*† |
| Creatine | 3.71  (2.61 – 4.57) | | 3.43  (2.16 – 4.52) | 4.52  (3.87 – 6.03)*†λ | 5.3  (3.91 – 7.98)*†λ | 4.66  (3.69 – 6) | 4.86  (4 – 6.32) | 5.2  (4.03 – 7.12) | 7.51  (5.52 – 10.8)*†λ |
| Creatinine | 3.22  (2.3 – 3.95) | | 3.14  (2.69 – 3.67) | 4.47  (3.71 – 5.36)*†λ | 4.16  (3.32 – 5)*†λ | 2.51  (1.96 – 3.37) | 2.83  (2.19 – 3.42) | 3.78  (2.36 – 4.44)* | 3.85  (3.04 – 4.96)*†λ |
| Glycine | 8  (7.04 – 9.04) | | 8.47  (7.4 – 10.4)* | 10  (8.45 – 12)*†λ | 9.88  (8.23 – 13.1)*†λ | 13.8  (12.6 – 15.8) | 14.3  (13 – 16) | 14.5  (12.6 – 17) | 16.2  (14.3 – 20.2)*† |
| L-Threonine | 1.54  (0 – 3.21) | | 0.75  (0 – 3.03) | 1.07  (0 – 3.09) | 0.92  (0 – 4.25) | 1.07  (0 – 3.79) | 1.06  (0 – 7.31) | 0.56  (0 – 4.86) | 2.08  (0 – 5.22) |
| D-Mannose | 1.25  (1.04 – 1.48) | | 1.03  (0.89 – 1.29)* | 1.78  (1.44 – 1.97)*†λ | 1.53  (1.26 – 1.72)*†λ | 0.79  (0.57 – 0.99) | 0.59  (0.42 – 0.87) | 0.94  (0.67 – 1.22) | 0.81  (0.51 – 1.08) |
| D-Glucose | 31  (24.8 – 35.8) | | 27.7  (20 – 36.2) | 37.2  (33.3 – 41.2) | 30.2  (23 – 36.7) | 26.7  (20.1 – 30.3) | 19.5  (13.9 – 26.1)* | 26.7  (21 – 31.9) | 21.2  (14.5 – 28.9) |
| L-Tyrosine | 1.17  (1.02 – 1.29) | | 1.2  (1.02 – 1.36) | 1.33  (1.1 – 1.48)*† | 1.42  (1.25 – 1.67)*†λ | 1.88  (1.7 – 2.15) | 1.92  (1.65 – 2.09) | 1.78  (1.62 – 2.08) | 2.05  (1.83 – 2.34)*† |
| L-Phenylalanine | 1.73  (1.54 – 1.91) | | 1.87  (1.65 – 2.13)* | 1.87  (1.76 – 2.18)*†λ | 2.17  (1.98 – 2.47)*†λ | 2.92  (2.69 – 3.14) | 2.87  (2.64 – 3.34) | 2.85  (2.56 – 3.28) | 3.08  (2.85 – 3.44)*† |
| 1-Methylhistidine | 1.15  (0.98 – 1.41) | | 1.19  (0.98 – 1.52) | 1.41  (1.16 – 1.61)*† | 1.34  (1.09 – 1.56)*† | 2.52  (2.21 – 2.79) | 2.44  (2.13 – 2.65) | 2.69  (2.43 – 2.94) | 2.46  (2.19 – 2.81) |

Data are median (interquartile range). Controls are normotensive pregnancies with appropriate growth for gestational age fetuses. FGR, fetal growth restriction; PE, preeclampsia; VLDL, Very low-density lipoprotein; IDL, Intermediate-density lipoprotein; LDL, Low-density lipoprotein; HDL, High-density lipoprotein; Glc/GalNAc, N-acetylglucosamine and N-acetylgalactosamine bonds.

* p<0.05 by Mann Whitney U test compared to controls.

† q<0.05 after 5% false discovery rate correction compared to controls.

λ p<0.05 by linear regression analysis adjusted for maternal body mass index, smoking, sample collection date, gestational age at delivery/sampling, route of delivery and fetal gender after 5% false discovery rate correction compared to controls.
